# The impact of varying levels of residual disease following cytoreductive surgery on survival outcomes in patients with ovarian cancer: a meta-analysis

**DOI:** 10.1186/s12905-024-02977-5

**Published:** 2024-03-15

**Authors:** Dana M. Chase, Anadi Mahajan, David Alexander Scott, Neil Hawkins, Linda Kalilani

**Affiliations:** 1 David Geffen School of Medicine, University of California, Los Angeles, CA USA; 2 Bridge Medical, London, UK; 3 GSK, Durham, NC USA

**Keywords:** Meta-analysis, Ovarian cancer, Residual disease, Cytoreductive surgery, Clinical outcomes

## Abstract

**Background:**

Residual disease following cytoreductive surgery in patients with ovarian cancer has been associated with poorer survival outcomes compared with no residual disease. We performed a meta-analysis to assess the impact of varying levels of residual disease status on survival outcomes in patients with ovarian cancer who have undergone primary cytoreductive surgery or interval cytoreductive surgery in the setting of new therapies for this disease.

**Methods:**

Medline, Embase, and Cochrane databases (January 2011 – July 2020) and grey literature, bibliographic and key conference proceedings, were searched for eligible studies. Fixed and random-effects meta-analyses compared progression and survival by residual disease level across studies. Heterogeneity between comparisons was explored via type of surgery, disease stage, and type of adjuvant chemotherapy.

**Results:**

Of 2832 database and 16 supplementary search articles screened, 50 studies were selected; most were observational studies. The meta-analysis showed that median progression-free survival and overall survival decreased progressively with increasing residual disease (residual disease categories of 0 cm, > 0–1 cm and > 1 cm). Compared with no residual disease, hazard ratios (HR) for disease progression increased with increasing residual disease category (1.75 [95% confidence interval: 1.42, 2.16] for residual disease > 0–1 cm and 2.14 [1.34, 3.39] for residual disease > 1 cm), and also for reduced survival (HR versus no residual disease, 1.75 [ 1.62, 1.90] for residual disease > 0–1 cm and 2.32 [1.97, 2.72] for residual disease > 1 cm). All comparisons were significant (*p* < 0.05). Subgroup analyses showed an association between residual disease and disease progression/reduced survival irrespective of type of surgery, disease stage, or type of adjuvant chemotherapy.

**Conclusions:**

This meta-analysis provided an update on the impact of residual disease following primary or interval cytoreductive surgery, and demonstrated that residual disease was still highly predictive of progression-free survival and overall survival in adults with ovarian cancer despite changes in ovarian cancer therapy over the last decade. Higher numerical categories of residual disease were associated with reduced survival than lower categories.

**Supplementary Information:**

The online version contains supplementary material available at 10.1186/s12905-024-02977-5.

## Background

Ovarian cancer is the fifth leading cause of cancer-related death among women in the USA [1]. Most cases of ovarian cancer are diagnosed at an advanced stage, for which the 5-year survival rate for patients with advanced disease is around 30% [2, 3]. Currently, the standard of care for treatment of advanced ovarian cancer includes surgery and platinum-based chemotherapy, followed by maintenance therapy [4]. Treatment regimens consist of either primary cytoreductive surgery with adjuvant platinum-based chemotherapy, or neoadjuvant chemotherapy with interval cytoreductive surgery and postoperative chemotherapy [4]. Both treatment options have similar outcomes and are typically selected based on patient and disease characteristics, with the latter being recommended for patients with bulky Stage III–IV disease who are poor surgical candidates or for whom there is a low likelihood of optimal cytoreduction [4, 5]. To achieve optimal outcomes, surgery for advanced ovarian cancer aims for tumour cytoreduction with no residual disease wherever possible [2], and chemotherapy aims to eradicate any microscopic disease remaining after surgery [6]. In the neoadjuvant chemotherapy setting, responsiveness to platinum-based chemotherapy may be known at the time of interval surgery and reflect disease status; while platinum sensitivity may alone be a prognostic factor, in the case of primary surgical debulking, the level of platinum sensitivity or resistance is unknown at the time of the procedure [7, 8]. Residual disease status after cytoreductive surgery for advanced-stage ovarian cancer is defined by the diameter of the residual tumour and is one of the most important prognostic factors for disease progression [7, 9, 10]. Residual disease status is commonly categorised as complete tumour resection or ‘no residual disease’ (residual disease 0 cm [R0]); ‘optimal cytoreduction’ (residual disease > 0–1 cm [R1]); or ‘suboptimal cytoreduction’ (residual disease > 1 cm [R2]) [9, 11–13].

Prior meta-analyses have shown that residual disease following primary cytoreductive surgery is associated with poorer survival outcomes in ovarian cancer compared with no residual disease, with a continuum of benefit observed across residual disease levels, R0–R2 [14–16]. Better overall survival was reported in patients with residual disease 0 cm versus ≤ 1 cm [14], in those with residual disease 0 cm or ≤ 2 cm versus those with residual disease > 2 cm [15], and in patients with residual disease < 1 cm versus residual disease > 1 cm following primary cytoreductive surgery [16]. However, with advancements in ovarian cancer management and treatment options in recent years [2], including utilisation of targeted treatments, a meta-analysis of studies published since 2011 is warranted to assess the impact of residual disease on patient outcomes.

The aim of this meta-analysis was to reassess the impact of varying levels of residual disease status on progression-free survival and overall survival specifically in patients with ovarian cancer who have undergone primary cytoreductive surgery or interval cytoreductive surgery in the setting of new therapies for ovarian cancer. This meta-analysis used data from publications previously identified in a recently published systematic literature review [17].

## Results

### Studies

Of 2832 screened articles from the original database search plus 16 from supplementary searches, 52 publications reporting on 50 primary studies were included (Supplementary Table 1) [10, 18–65]. This comprised 43 observational studies (41 retrospective [10, 18–28, 30, 31, 33–35, 37–39, 41, 42, 44–52, 54, 56–58, 60, 62–65], 2 prospective [32, 61]), 4 retrospective analyses of RCTs [40, 43, 53, 55], and 3 RCTs [29, 36, 59]. Included studies were conducted either solely or partly (for multinational studies) in Australia, Austria, Belgium, Canada, China, Denmark, Finland, France, Germany, Italy, Japan, the Netherlands, New Zealand, Norway, Republic of Korea, Spain, Sweden, United Kingdom, and the United States. Data on progression-free survival per residual disease status were available from 21 studies (2 randomised controlled trials [29, 36], 15 observational studies [23–25, 27, 31–35, 44, 45, 49, 51, 56, 58, 61], and 4 retrospective analyses of randomised controlled trials [40, 43, 53, 55]) and data on overall survival per residual disease status were available from 48 studies (3 randomised controlled trials [29, 36, 59], 4 retrospective analyses of randomised controlled trials [40, 43, 53, 55], and 41 observational studies [10, 18–28, 30, 31, 33–35, 37–39, 41, 42, 44–52, 54, 56–58, 61–65]). There was substantial heterogeneity in patient baseline characteristics and reported variables across the studies. The PRISMA flow chart of included publications and key characteristics of each publication included in the systemic literature review have been described previously [17].

Briefly, over half (*n* = 29) of the studies included in this analysis were published between 2016 and 2020, with the remaining studies published between 2011 and 2015. Nearly a third of all studies (*n* = 18, 36%) included patients who had undergone combination surgery (either primary and interval cytoreductive surgery [*n* = 16], or primary and interval cytoreductive surgery plus hyperthermic intraperitoneal chemotherapy [*n* = 2]) [22, 25, 27, 28, 30, 32–35, 42, 45, 47, 48, 57, 60–63]; 15 and 4 studies included patients who had primary cytoreductive surgery [10, 18–21, 23, 31, 36, 37, 39, 41, 44, 49, 55, 57] or interval cytoreductive surgery [26, 50, 58, 64] only, respectively; and 13 reported cytoreductive surgery without specifying surgery type [24, 25, 29, 38, 40, 43, 46, 51–54, 59, 65]. The majority of studies (*n* = 34, 68%) enrolled patients with Stage III/IV disease only [10, 19, 20, 22, 23, 26–30, 33, 34, 37, 39–42, 44–51, 53–56, 58, 59, 62, 64, 65]; 16 studies included patients with mixed Stages I–IV [18, 21, 24, 25, 31, 32, 35, 36, 38, 43, 52, 57, 60, 61, 63] and only 2 studies among these reported data for Stage I/II and Stage III/IV separately [25, 66]. Chemotherapy in the adjuvant setting was reported in 25 studies whilst 4 studies reported chemotherapy use in the neoadjuvant setting. Additionally, 18 studies reported using both neoadjuvant and adjuvant chemotherapy; usage of neoadjuvant and adjuvant chemotherapy was not reported in 3 studies. Due to the wide range of interventions used in the adjuvant and neoadjuvant setting, comparisons across treatments were not feasible.

### Reporting of study measures: progression-free survival, overall survival, and residual disease status

The progression-free survival and overall survival definitions varied across studies [17]. Across all studies, residual disease status was defined as no residual disease (0 cm), optimal cytoreduction (residual disease measuring > 0–1 cm), and suboptimal cytoreduction (residual disease measuring > 1 cm). Optimal cytoreduction was defined using a variety of approaches across studies (residual disease 0.1–1 cm; residual disease 0.01–1 cm; residual disease 0–1 cm; residual disease > 0–1 cm; residual disease < 1 cm); these were all categorised as residual disease > 0–1 cm for the purposes of this meta-analysis. In 4 of these studies [24, 25, 35, 52] optimal cytoreduction also included patients with no residual disease. An additional category, residual disease > 2 cm, was also reported for overall survival in 3 studies [10, 37, 62].

### Relationship between residual disease and survival

**Fig. 1:**
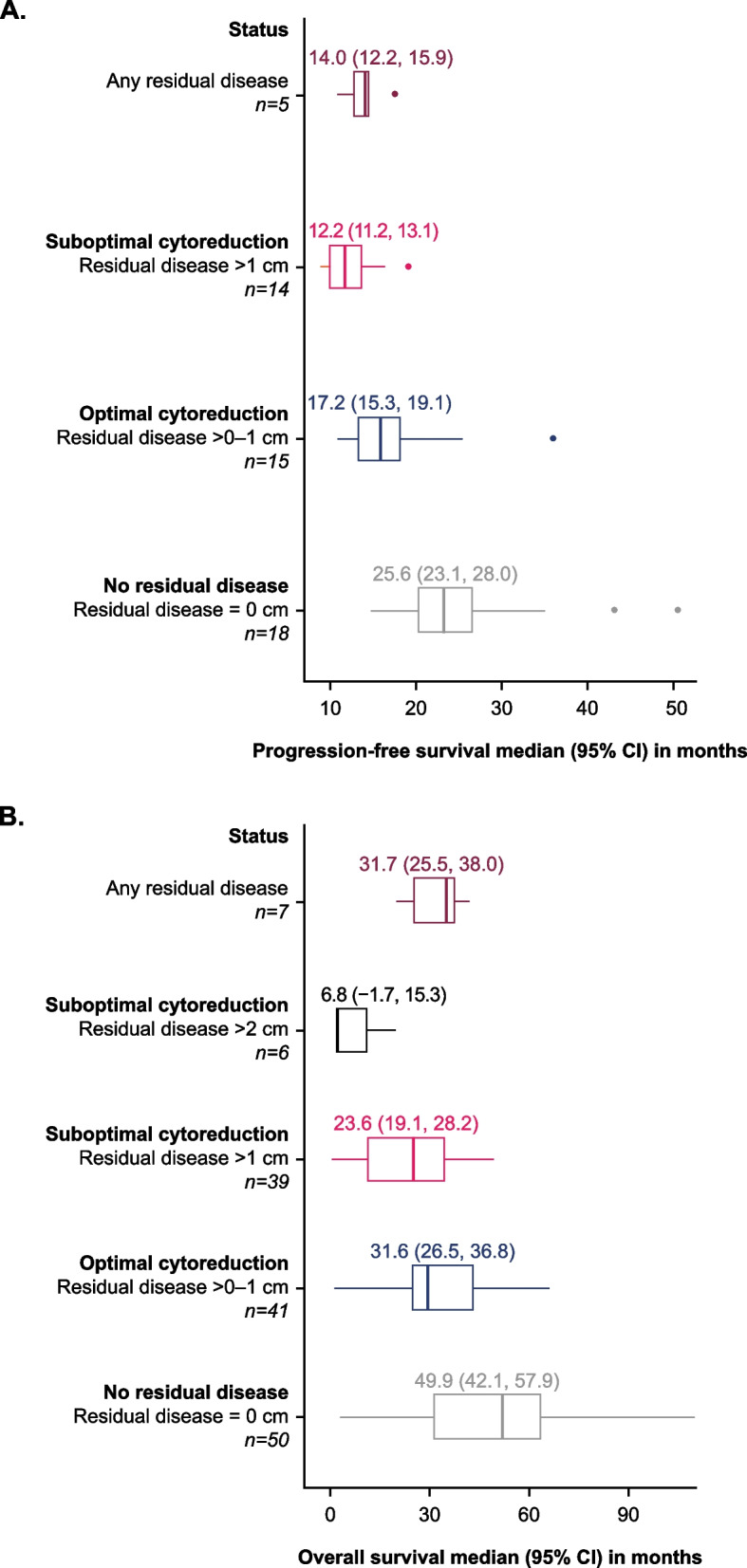
Pooled median progression-free survival **A** and overall survival **B** by residual disease status across studies. N numbers represent number of data points used in analysis. Median progression-free survival per residual disease status was reported in 12 studies and median overall survival per residual disease status was reported in 27 studies. Residual disease > 1 cm category includes studies reporting residual disease 1–2 cm and residual disease > 1 cm. CI, confidence interval

Median progression-free survival ranged from 9 months to 50.2 months and median overall survival ranged from 6 to 110 months across studies. When analysed by residual disease category, median progression-free survival and overall survival for ‘no residual disease’ (residual disease = 0 cm) was longer than for ‘any residual disease’ (residual disease >0 cm; Fig. 1A) and both median progression-free survival and median overall survival decreased across progressively higher residual disease categories. The median pooled progression-free survival (95% confidence interval [CI]) for the no residual disease category was 25.6 (23.1, 28.0) months, compared with significantly worse median pooled progression-free survival of 17.2 (15.3, 19.1) months for residual disease >0–1 cm, 12.2 (11.2, 13.1) months for residual disease >1 cm, and 14.0 (12.1, 15.9) months for any residual disease (*p*<0.05; Fig. 1A). Median pooled overall survival (95% CI) for the no residual disease category was 49.9 (42.1, 57.9) months, whereas all residual disease categories were significantly shorter; 31.6 (26.5, 36.8) months for residual disease >0–1 cm, 23.6 (19.1, 28.2) months for residual disease >1 cm, 6.8 (−1.7, 15.3) months for residual disease >2 cm, and 31.7 (25.5, 38.0) months for any residual disease (*p*<0.05; Fig. 1B).

**Fig. 2:**
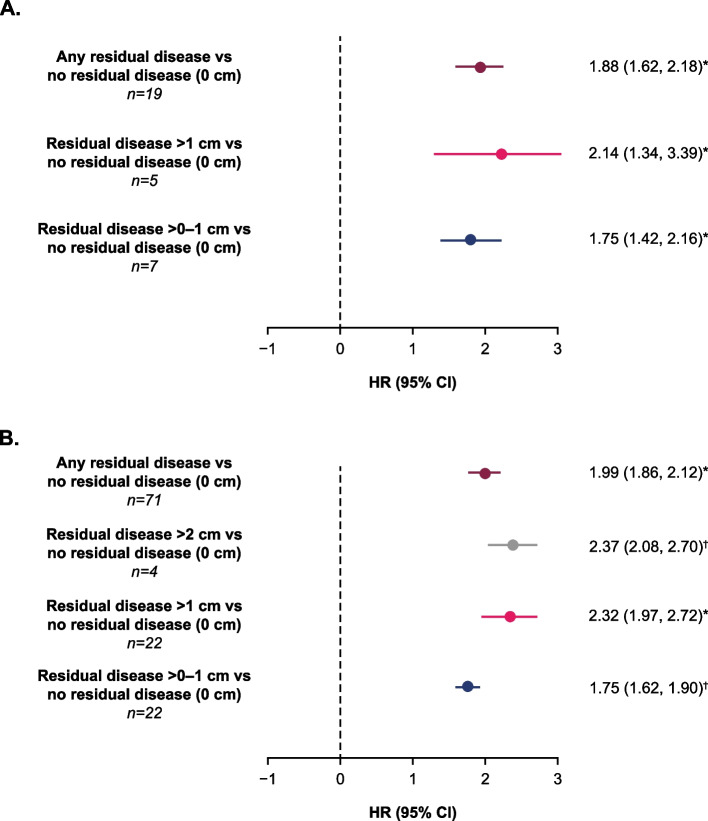
Pooled HRs for progression-free survival **A** and overall survival **B** by residual disease status. *Random effects. †Fixed effects. Residual disease > 1 cm category includes studies reporting residual disease 1–2 cm and residual disease > 1 cm. All comparisons were considered significant (*p* < 0.05). CI, confidence interval; HR, hazard ratio

For studies that calculated the hazard ratio (HR) comparing progression-free survival and overall survival across residual disease categories, and adjusting for baseline characteristics on a study-by-study basis, HRs showed that lower residual disease categories were associated with improved progression-free survival and overall survival compared with higher residual disease categories (Fig. 2). The pooled HR for disease progression for any residual disease compared with no residual disease was 1.88 (95% CI 1.62, 2.18) and for reduced survival was 1.99 (95% CI 1.86, 2.12). The increased risk of earlier progression or death associated with residual disease compared with no residual disease became more pronounced with increasing residual disease: HRs versus no residual disease for disease progression were 1.75 (95% CI: 1.42, 2.16) for residual disease > 0–1 cm and 2.14 (95% CI 1.34, 3.39) for residual disease > 1 cm; for reduced survival, HRs versus no residual disease were 1.75 (95% CI 1.62, 1.90) for residual disease > 0–1 cm, 2.32 (95% CI 1.97, 2.72) for residual disease > 1 cm, and 2.37 (95% CI 2.08, 2.70) for residual disease > 2 cm. All comparisons were significant at *p* < 0.05.

### Subgroup analyses

**Fig. 3:**
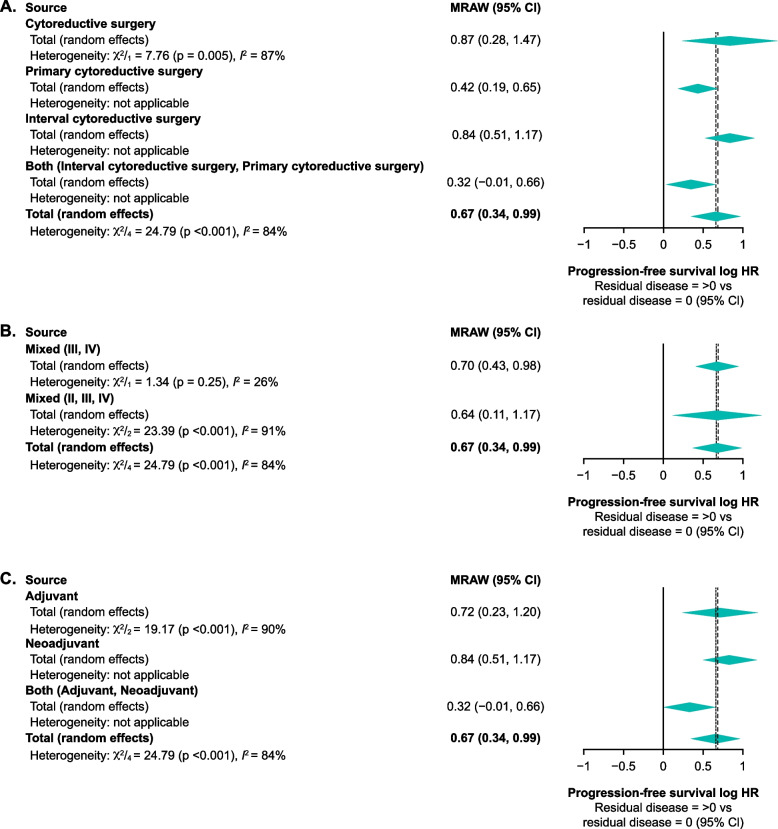
Progression-free survival by type of surgery **A** disease stage **B** and chemotherapy received **C**. Log HR for residual disease > 0 vs residual disease = 0. Thick dotted lines represent fixed effects and thin dotted lines represent random effects for the total effect size. CI, confidence interval; HR, hazard ratio; MRAW, raw or untransformed mean

Subgroup analysis showed the same pattern of association between residual disease and median progression-free survival/overall survival, with pooled median progression-free survival and overall survival estimates decreased for higher residual disease categories compared with lower categories. A random effects model was considered appropriate, due to substantial heterogeneity (I^2^ ≥ 50) reported across the studies. Within type of surgery, disease stage, and type of adjuvant chemotherapy subgroups, progression-free survival was significantly worse with higher residual disease versus no residual disease categories, with no statistically significant differences between subgroups for type of surgery (Fig. 3A; *p* = 0.07), disease stage (Fig. 3B; *p* = 0.83), or adjuvant chemotherapy type (Fig. 3C; *p* = 0.09). Analyses of progression-free survival HRs by residual disease > 0 cm versus no residual disease are shown in Supplementary Fig. 1.

**Fig. 4:**
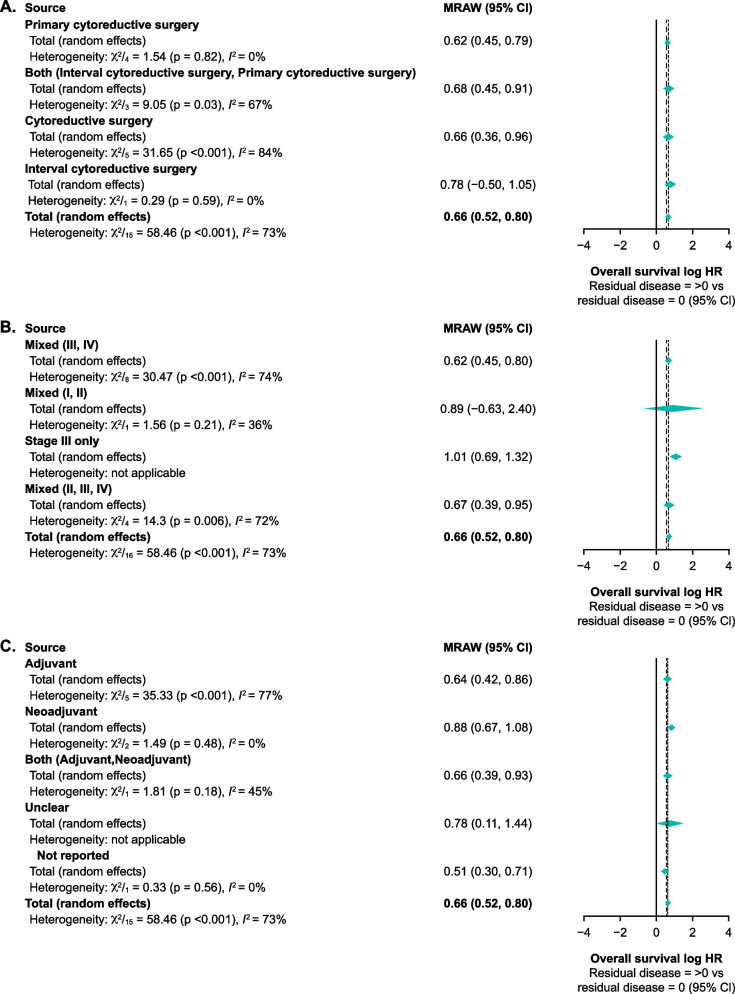
Overall survival by type of surgery **A** disease stage **B** and chemotherapy received **C** Log HR for residual disease > 0 vs residual disease = 0. Thick dotted lines represent fixed effects and thin dotted lines represent random effects for the total effect size. CI, confidence interval; HR, hazard ratio; MRAW, raw or untransformed mean

The corresponding subgroup analyses for overall survival per residual disease status are shown in Fig. 4. There was a statistically significant decrease in overall survival with residual disease > 0 cm versus no residual disease categories irrespective of subgroup, with no statistically significant differences between subgroups for type of surgery (Fig. 4A; *p* = 0.82), disease stage (Fig. 4B; *p* = 0.21), or adjuvant chemotherapy type (Fig. 4C; *p* = 0.17). Analyses of overall survival HRs by residual disease > 0 cm versus no residual disease are shown in Supplementary Fig. 2.

## Discussion

### Summary of main results

This meta-analysis provides an update on the impact of residual disease status on survival outcomes following primary cytoreductive surgery or interval cytoreductive surgery as a first-line treatment for ovarian cancer. In our analysis, residual disease status was highly predictive of overall survival and progression-free survival, with higher levels of residual disease associated with increased disease progression and reduced survival compared with lower levels of residual disease, and particularly versus no residual disease. Subgroup analyses revealed that the relationship between residual disease and progression-free survival/overall survival was not affected by the type of surgery, disease stage, or type of chemotherapy.

### Results in the context of published literature

Previous meta-analyses investigating the impact of residual disease in ovarian cancer have reported improved survival in patients with no/lower residual disease compared with patients who had higher residual disease [14–16]. In addition, a systematic review assessing survival outcomes in relation to residual disease status following interval cytoreductive surgery reported that the best survival outcomes were in patients with no macroscopic residual disease [67]. In this meta-analysis, we report on the impact of residual disease on survival outcomes in the setting of new therapies for ovarian cancer and showed that no residual disease confers the best progression-free survival and overall survival outcomes in patients with advanced ovarian cancer undergoing primary cytoreductive surgery or interval cytoreductive surgery. As such, a combination of tumour biology, disease distribution, and surgical approach are strong determinants of patient outcomes despite the introduction of new therapies.

### Implications for practice and future research

The first period between initial diagnosis and recurrence is typically the longest treatment-free interval, with progression-free survival and overall survival becoming increasingly shorter after each line of treatment [68]. If residual disease can help predict that treatment-free interval, it could be used to determine which patients have the highest risk of earlier progression or death so that healthcare providers can tailor intensive monitoring and/or therapy accordingly. Following on, it would then be informative to investigate how a patient’s known residual disease status influences management decisions. In the primary cytoreductive surgery setting, the patient’s likely response to platinum-based chemotherapy is unknown at the time of surgery [69], and their subsequent residual disease status is likely affected by the disease biology and/or distribution. Conversely, with interval cytoreductive surgery, the patient’s residual disease status may be affected by their prior response to platinum-based chemotherapy [69]. Not only does this highlight the need for markers of chemoresistance [70], but future studies could evaluate residual disease status in response to chemotherapy as well as after primary cytoreductive surgery/interval cytoreductive surgery, to ascertain if such data could inform prognostic risk and management. Also, as residual disease status could potentially inform prognosis [69, 70], it could be used alongside other known risk factors to categorise patients as having low or high risk ovarian cancer. Moreover, cost–benefit analyses based on residual disease status may help to clarify if both high- and low-risk patients are eligible for maintenance therapy (if both high- and low-risk patients by residual disease status show similar benefit) or if a different strategy is needed for higher-risk patients. Ultimately, adopting a more personalised treatment approach is warranted, which takes residual disease-associated risk into account, alongside other prognostic factors including patient characteristics [71, 72], disease stage [5, 72], and tumour mutational burden [73]. The findings from this meta-analysis highlight the importance of residual disease in survival outcomes; a greater awareness of the potential of residual disease as a prognostic factor could aid disease management and thus contribute towards improving individual patient care.

### Strengths and weaknesses

Although an adequate number of studies were included overall, limitations of our study include the predominance of data from observational studies. Comparison across treatments was not feasible due to the wide range of interventions used in adjuvant and neoadjuvant settings, and there were differences in how residual disease was defined across the studies. Availability of data on diagnosis, disease stage, and histology was relatively limited, restricting in-depth analysis of the impact of these factors on the overall results. Similarly, there were only limited data for factors affecting overall survival and progression-free survival with regard to residual disease status; any subtle differences in the effects of disease stage, type of cytoreductive surgery, and type of adjuvant chemotherapy on residual disease status could not be discerned, limiting any robust conclusions. Where available, data were stratified by known prognostic factors and these results were consistent with overall results; however, due to a limited number of studies for each subgroup analyses, multivariate analysis of factors was not possible. The considerable heterogeneity in data between studies – as shown by the high I^2^ values in Figs. 3 and 4 – was a likely consequence of variations in study types, design, and populations included in the meta-analysis. This was, however, partly mitigated by the use of random-effects models when I^2^ values were 50% or greater. Taken together, these limitations may reduce the generalisability of the study findings to clinical practice.

Another important factor to consider when interpreting these data is the ever-evolving ovarian cancer treatment landscape [74]. Introduction of maintenance therapies in the ovarian cancer treatment landscape has greatly improved patient outcomes [75–78]. Bevacizumab was first approved as a first-line maintenance therapy for patients with ovarian cancer, in Europe in November 2011 and in the United States in June 2018 [76, 77]. Poly-(ADP) ribose polymerase (PARP) inhibitors were then introduced as first-line maintenance therapies in the United States in December 2018 and in Europe in January 2019, and have significantly increased progression-free survival for patients with ovarian cancer [75, 78, 79]. Over half (*n*=29) of the studies included in this analysis were published between 2016 and 2020, coinciding with the introduction of maintenance therapies in the ovarian cancer treatment landscape. The remaining studies (*n*=23), however, pre-date the approval of PARP inhibitors, so the standard of care would have included cytoreductive surgery and platinum-based chemotherapy only [80]. It is possible that these maintenance therapies had considerable effects on PFS and OS, and could partially explain the high heterogeneity observed in this analysis as well as impacting the generalisability of the findings. Despite the introduction of maintenance therapies, residual disease status remained a strong predictor of patient outcomes. Future analysis is warranted to examine the effects of maintenance therapies on PFS and OS in relation to residual disease.

## Conclusions

This meta-analysis demonstrated that higher residual disease categories, when compared with lower categories, were highly predictive of worse overall survival and progression-free survival in adults with ovarian cancer who have undergone primary cytoreductive surgery or interval cytoreductive surgery, despite changes in the treatment landscape of ovarian cancer over the last decade.

## Methods

### Systematic literature review data extraction

As previously described [17], a structured literature search of MEDLINE, Excerpta Medica Database (Embase) and Cochrane CENTRAL databases was conducted on July 7, 2020 (with date limits from January 1, 2011 to July 7, 2020), and was supplemented by searches of grey literature, bibliographic sources, and conference proceedings (American Society of Clinical Oncology, European Society for Medical Oncology, Society of Gynecologic Oncology 2019–2020) carried out between August 14 and August 20, 2020, identifying relevant publications over the previous 10 years (2011 to 2020). Full texts were obtained after assessing titles and abstracts for relevance. A comprehensive search strategy based on the population, intervention, comparator, outcome, and study type framework was used to evaluate search results. Key eligibility criteria for studies were clinical trials or observational studies involving > 200 adult women (typically ≥ 18 years) with ovarian cancer (including fallopian tube cancer and primary peritoneal cancer) where overall survival and progression-free survival were evaluated per residual disease status after primary cytoreductive surgery or interval cytoreductive surgery. Case studies and case reports were excluded.

Two independent reviewers assessed each citation for eligibility, with discrepancies resolved by a third reviewer. This same process was used for data extraction from eligible studies. Study quality was assessed by recommended instruments for randomised controlled trials [81] and observational studies [82] as previously described [17], with all included studies being fair quality or above.

Definitions for progression-free survival and overall survival were based on individual study definitions, and varied across studies [17]. Full systematic literature review details are reported per PRISMA guidelines in the prior systematic review publication [17].

### Statistical analysis

Analyses were conducted in R (The R Foundation, Vienna, Austria). Pooled HRs and 95% CI were calculated from individual study HRs using a linear regression model, to determine any association between residual disease and progression-free survival or overall survival. Heterogeneity was assessed by the I^2^ statistic. Random effects were used when the I^2^ was 50% or greater; otherwise, fixed effects were used to compare progression-free survival and overall survival by residual disease levels across all studies. Residual disease levels were categorised as follows: > 0 cm versus 0 cm; > 0–1 cm versus 0 cm; > 1 cm versus 0 cm, and (for overall survival only) > 2 cm versus 0 cm. Pooled analyses that included more than two studies with residual disease categories were reported.

Subgroup analyses were performed to examine progression-free survival and overall survival by the following clinical characteristics: type of surgery (primary cytoreductive surgery; either interval cytoreductive surgery or primary cytoreductive surgery; cytoreductive surgery; and interval cytoreductive surgery), disease stage (mixed [I, II]; mixed [II, III, IV]; stage III only; mixed [III, IV]), and type of chemotherapy (adjuvant; neoadjuvant followed by adjuvant; or studies that reported both). All comparisons were considered significant at *p* < 0.05.

### Supplementary Information

**Supplementary Material 1.**

## Data Availability

GSK makes available anonymised individual participant data and associated documents from interventional clinical studies that evaluate medicines, upon approval of proposals submitted to https://www.gsk-studyregister.com/en/.
