# Supplementary material for: The impact of varying levels of residual disease following cytoreductive surgery on survival outcomes in patients with ovarian cancer: a meta-analysis
Source: BMC Womens Health. 2024 Mar 15;24:179. doi: 10.1186/s12905-024-02977-5 (PMC10941390; doi:10.1186/s12905-024-02977-5)
Supplement: Supplementary file 1 — Supplementary Material 1. [file 12905_2024_2977_MOESM1_ESM.docx]

# Supplementary Materials

## Supplementary Table 1. Publications included in meta-analysis

| Publications | Reference |
| --- | --- |
| Ataseven 2014 | [18] |
| Ataseven 2016 | [19] |
| Ataseven 2018 | [20] |
| Braicu 2011 | [21] |
| Bristow 2011 | [22] |
| Chang 2012 | [23] |
| Chen 2014 | [24] |
| Cheng 2020a | [25] |
| Davidson 2019 | [26] |
| Delga 2020 | [27] |
| Deng 2017 (JICR)^†^ | [35] |
| Deng 2017 (MDACC)**^†^** | [35] |
| Di Giorgio 2017 | [28] |
| Fader 2013 (GOG protocol 182) | [29] |
| Fagö-Olsen 2014 | [30] |
| Feng 2016 | [31] |
| Fleming 2018 | [32] |
| Gadducci 2017 | [33] |
| Gao 2019 | [34] |
| González Martín 2019 (ICON7 Trial) | [36] |
| Hosono 2011 | [37] |
| Kalapotharakos 2012 | [38] |
| Kumar 2016 | [39] |
| Landrum 2013 | [40] |
| Langstraat 2011 | [41] |
| Luyckx 2012 | [42] |
| Mahner 2013 | [43] |
| Manning-Geist 2018 | [44] |
| Markauskas 2014 | [45] |
| Melamed 2017 | [46] |
| Mizuno 2015 | [47] |
| Munoz-Casares 2016 | [48] |
| Phelps 2017 | [49] |
| Phillips 2018 | [50] |
| Ren 2015 | [51] |
| Ren 2017 | [52] |
| Rodriguez 2013 (GOG 182) | [53] |
| Rosendahl 2018 | [54] |
| Rungruang 2017 | [55] |
| Rutten 2015 | [56] |
| Rutten 2017 | [57] |
| Searle 2020 | [58] |
| Sørensen 2019 | [10] |
| Tewari 2016 (GOG protocol 0218) | [59] |
| Timmermans 2018 | [60] |
| Trillsch 2013 | [61] |
| Trope 2012 | [62] |
| Van Altena 2013 | [63] |
| Vincent 2020 | [64] |
| Zhou 2018 | [65] |

^*^These references report on the same study.

†These references report on the same study.

##
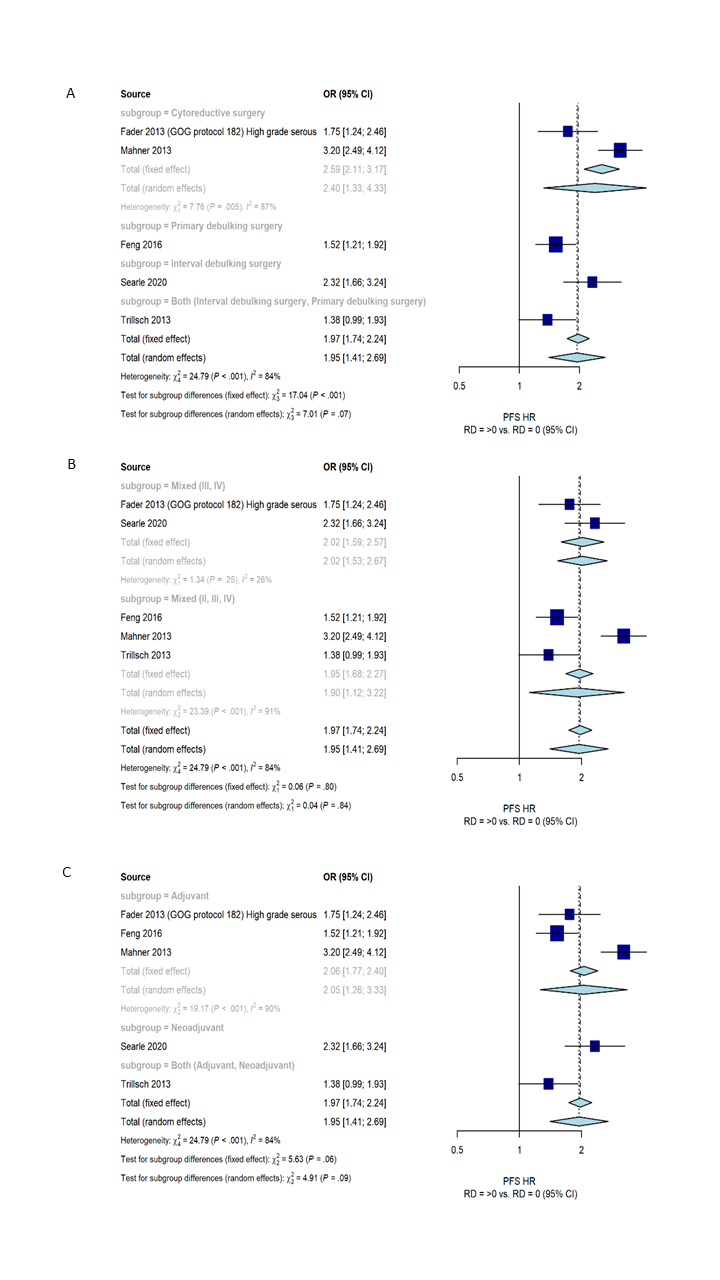
Supplementary Figure 1. Progression-free survival (HR for residual disease >0 vs residual disease=0) by type of surgery (A), disease stage (B), and type of chemotherapy received (C)

Thick dotted lines represent fixed effects and thin dotted lines represent random effects for the total effect size.

These analyses report random effects across all subgroups irrespective of *I^2^.*

CI, confidence interval; HR, hazard ratio; OR, odds ratio; PFS, progression-free survival; RD, residual disease.

##
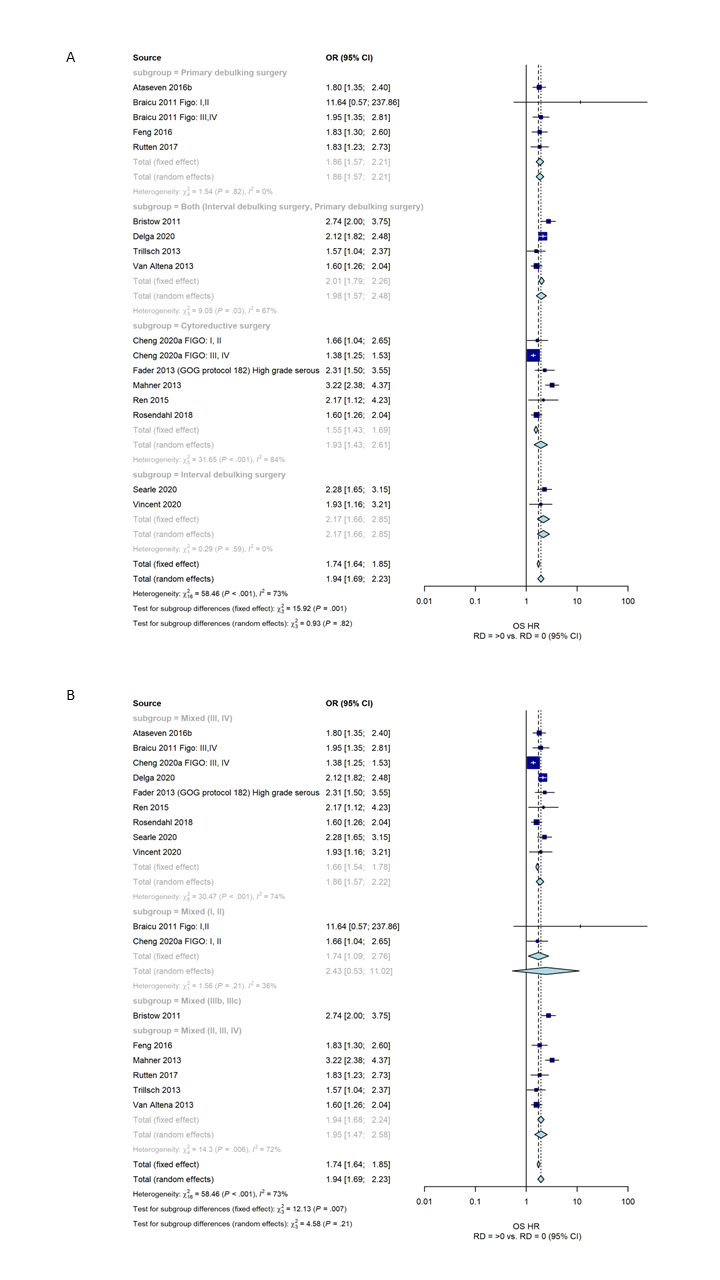
Supplementary Figure 2. Overall survival (HR for residual disease>0 vs residual disease=0) by type of surgery (A), disease stage (B), and type of chemotherapy received (C)


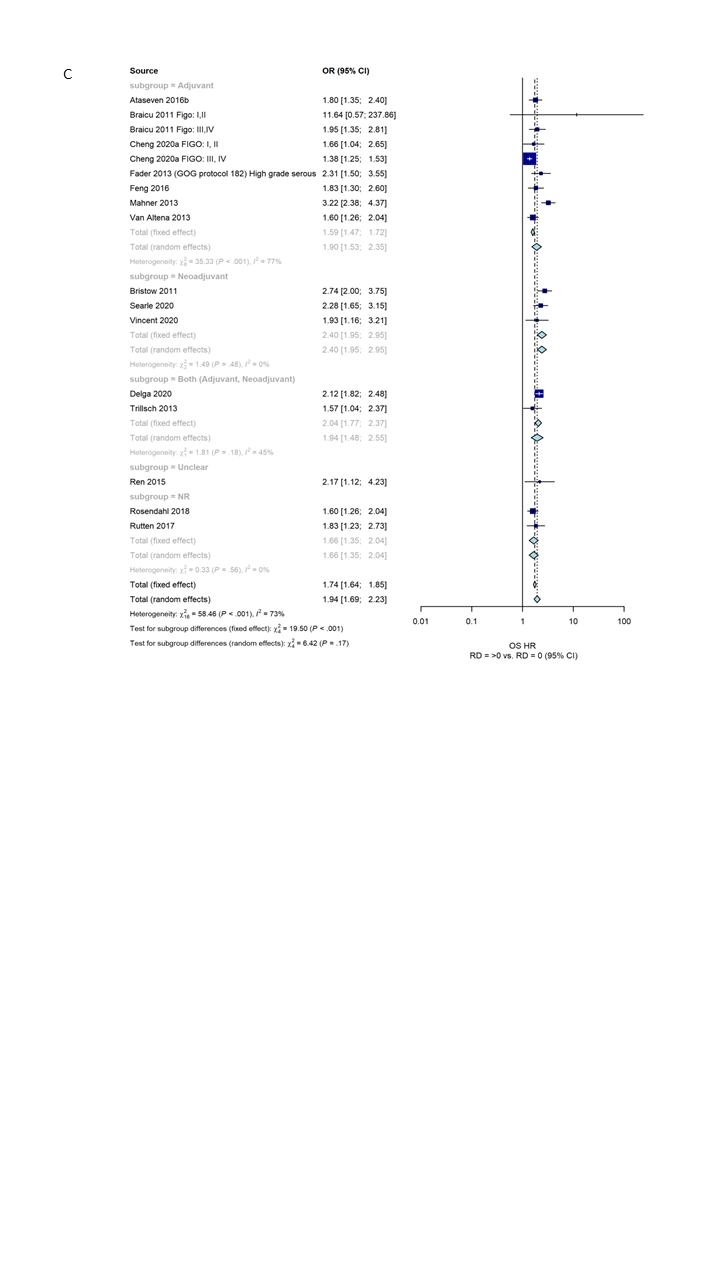


Thick dotted lines represent fixed effects and thin dotted lines represent random effects for the total effect size.

These analyses report random effects across all subgroups irrespective of *I^2^.*

CI, confidence interval; HR, hazard ratio; MRAW, raw or untransformed mean; OS, overall survival; RD, residual disease.
